# Counselling versus Low-Intensity Cognitive Behavioural Therapy for persistent sub-threshold and mild Depression (CLICD): study protocol for a pilot/feasibility randomised controlled trial

**DOI:** 10.1186/2193-1801-3-654

**Published:** 2014-11-05

**Authors:** Elizabeth Freire, Jill Morrison, Christopher Williams, Mick Cooper, Robert Elliott, Alex McConnachie, Andrew Walker, Deborah Heard

**Affiliations:** School of Education, University of Aberdeen, MacRobert Building, King’s College, Aberdeen, AB25 5UA UK; Institute of Health and Wellbeing, University of Glasgow, General Practice and Primary Care, 1 Horselethill Road, Glasgow, G12 9LX UK; University of Glasgow, Institute of Health and Wellbeing, Administration Building, Gartnavel Royal Hospital, 1055 Great Western Road, Glasgow, G12 0XH UK; Department of Psychology, University of Roehampton, Holybourne Avenue, London, SW15 4JD UK; Counselling Unit, School of Psychological Sciences & Health, University of Strathclyde, GH507 Graham Hills Building, 40 George Street, Glasgow, G1 1QE UK; Robertson Centre for Biostatistics, University of Glasgow, Boyd Orr Building, University Avenue, Glasgow, G12 8QQ UK

**Keywords:** Depression, Primary care, Person-centred counselling, Low intensity cognitive behavioural therapy, Randomised controlled trial, Pilot study

## Abstract

**Background:**

Persistent sub-threshold depressive symptoms are important because almost all patients who experience symptoms for more than 2 years go on to develop major depressive episodes. The National Institute for Health and Care Excellence in the United Kingdom recommends research into the efficacy of person centred counselling and low-intensity cognitive behavioural therapy for persistent sub-threshold and mild depression.

**Methods/design:**

A two-arm, parallel group, pilot randomised trial to test the key components of trial delivery. The participants will be 50 patients with the diagnosis of persistent sub-threshold depressive symptoms and mild depression, recruited at five general practices in Glasgow, Scotland. Eligible patients will be randomised to receive either Person-Centred Counselling (PCC) or Low-Intensity Cognitive Behavioural Therapy (LI-CBT). The primary outcome measures are recruitment, adherence and retention rates at six months from baseline. The secondary outcome measures are changes at 6 months on GRID-HAMD-17; recovery from, or prevention of, depression according to DSM-IV diagnosis at 6 months; changes at 6 months on the PHQ-9, WSAS, EQ5D, and SF12v2 MH Enhanced.

We will provide estimates, with adequate precision, to help design future studies, of the recruitment rate and the proportion followed-up at 6 months; and identify potential moderators of outcome.

**Discussion:**

Evidence of comparative effectiveness of commonly used psychological treatments such as person-centred counselling and low intensity cognitive behavioural therapy is lacking in patients with sub-threshold and mild depression. This study will provide the information needed to construct a trial comparing these two treatments. This would help to inform early intervention treatment strategies for these conditions.

**Trial registration:**

Current Controlled Trials ISRCTN Register ID: ISRCTN60972025

## Introduction

Depression is a major public health problem that causes significant costs to individuals and society (Cassano and Fava 2002). It is associated with poor quality of life, impaired interpersonal and family relationships, occupational disadvantage, residual disability, and suicide (Thornicroft and Sartorius 1993). Almost 800,000 consultations for psychological signs and symptoms (mainly depression and/or anxiety) were conducted in general practice in Scotland in 2012 – 13 (Information and Statistics Division Scotland 2013). Persistent depressive symptoms below the threshold for major depression cause chronic illness and have a high risk of progression (Goldberg and Huxley 1992). Almost all patients with sub-threshold depressive symptoms lasting longer than 2 years eventually develop major depressive episodes (Klein et al. 2000).

### Rationale for the Study

The National Institute for Health and Care Excellence (NICE) (National Institute for Clinical Excellence 2009) recommends establishing if counselling and low-intensity cognitive behavioural interventions are effective alternatives to treatment as usual, in the management of persistent sub-threshold depressive symptoms and mild depression. Depressive symptoms are ‘persistent’, if they continue despite active monitoring and/or low-intensity intervention, or have been present for a considerable time. NICE recommends a non-inferiority trial design, using both observer and patient-rated assessments of improvement, and reporting short and medium-term outcomes of at least 18 months duration. The Scottish Intercollegiate Guidelines Network (SIGN) similarly recommends research on the long-term effectiveness of non-pharmaceutical interventions for depression (Scottish Intercollegiate Guidelines Network 2010).

Person-centred counselling (PCC), also known as non-directive therapy or Rogerian therapy, is the most common psychological intervention offered in community settings in the UK (Mellor-Clark et al. 2001). PCC focuses on providing an empathic, accepting, and genuine therapeutic relationship, which aims to foster clients’ inner capacities and resources, promoting positive change (Rogers 1951). Evidence, from a number of trials in the UK comparing counselling with usual GP care for patients with depression, suggests that counselling is superior to standard GP care with an effect size of 0.5 to 0.6, with the strongest effects in the short term (Bower and Rowland 2006).

Low intensity Cognitive Behavioural Therapy (CBT) guided self-help is recommended by NICE and SIGN for mild to moderate depression. Guided self-help CBT is considered a low intensity (LI) intervention because the amount of practitioner time is limited, compared to traditional high intensity (HI) expert-led treatments. Guided self-help CBT can be delivered through books, classes, computers and online resources (Williams and Morrison 2010). A review of the efficacy of treatments for depression found the overall effect of guided CBT self-help interventions was 0.8, (95% CI 0.58 – 1.01) with improved results when support or guidance was provided (Gellatly et al. 2007). A recent meta-analysis suggested equivalent outcomes for expert delivered CBT and for guided CBT self-help for depression (Cuijpers et al. 2010).

NICE also recommends assessment of the acceptability of the treatment options and the investigation of moderator variables affecting outcomes. A recent meta-analysis of patients’ preferences found that patients who were matched to their preferred talking therapy were less likely to drop out of therapy and had better outcomes (Swift et al. 2011). There is also a small but significant positive effect of patient prior expectation on successful treatment outcome (Constantino et al. 2011). In addition, about 85% of adults with depression experience significant symptoms of anxiety and their presence results in less positive treatment outcomes (Gorman 1997). Finally, patients with high alexithymia (i.e., difficulty in perceiving, differentiating, and expressing emotions) obtain less favourable outcomes from psychological therapies (McCallum et al. 2003).

## Methods/design

### Study Aims

This study aims to test the feasibility of a randomised controlled trial of the clinical and cost effectiveness of Low Intensity Cognitive Behaviour Therapy (LI-CBT) and Person-Centred Counselling (PCC) for patients with persistent sub-threshold depressive symptoms and mild depression. It will provide estimates for the recruitment, adherence, and retention rates, as well as estimates of the variability of outcome measures to inform power calculations for a definitive trial. Estimates of comparative intervention effects will be produced, but this study is not designed to have sufficient power to detect differences, nor to demonstrate non-inferiority between interventions. It will also:

- Test the feasibility of measuring alexithymia, anxiety, preference for treatment, and outcome expectations as potential moderators of outcomes.
- Identify other factors that are potential moderators of outcomes.
- Test the feasibility of carrying out adherence/competence checks of the treatments delivered.
- Identify the feasible and necessary data to collect for a cost-effectiveness comparative analysis.

It will consider a range of variables at baseline as possible moderators (e.g., chronicity and severity of depressive symptoms and demographic variables). Finally, this study will help to identify any potential problems that will need to be addressed before a definitive trial.

### Type of study

This study is a two-arm, parallel group, randomised trial that will be performed according to the Research Governance Framework for Health and Community Care (Second edition, 2006) (Department of Health 2005). A two-arm design was adopted rather than a three-armed study with a control/usual care comparator. This was decided after discussion with the funder who was concerned that a three arm trial would be unnecessarily complex with little chance of testing out moderator variables. A two–arm study would have lower cost and more power to compare the relative cost-effectiveness of the interventions.

### Research questions

#### Primary research question

What are the likely recruitment, adherence, and retention rates over six months for a trial comparing PCC with LI-CBT in primary care for persistent sub-threshold and mild depression?

#### Secondary research questions

1. What sample size would be required for a larger comparative study of short-term (6 months) outcomes between PCC and LI-CBT in primary care for persistent sub-threshold and mild depression?
2. What is the feasibility of measuring alexithymia, anxiety, preference for treatment, and outcome expectations as potential moderators of outcomes?
3. What other factors can be identified as potential moderators of outcomes?
4. What is the feasibility of carrying out adherence/competence checks of the treatments delivered?
5. What data are feasible and necessary to collect for a cost-effectiveness comparative analysis?

### Outcome measures

The primary outcome measures are recruitment, adherence and retention rates at six months from baseline.

The secondary outcome measures are:

1. Changes at 6 months on GRID-HAMD-17 (Williams et al. 2008)
2. Recovery from, or prevention of, depression according to DSM-IV (American Psychiatric Association 2000) diagnosis at 6 months;
3. Changes at 6 months on the Patient Health Questionnaire – 9 (PHQ-9) (Spitzer et al. 1999), Work and Social Adjustment Scale (WSAS) (Mundt et al. 2002), Euroquol (EQ5D) (The EuroQuol Group 1990), and SF12v2 MH Enhanced (Mariush and Turner-Bowker 2009

Evaluations at baseline, 3 and 6 months will all be conducted in person between the research assistant and subject and are summarised in Table 1.

**Table 1:** **Measures taken during the study**

Screening	Baseline	3 months	6 months
• PHQ-9.	• SCID interview	• PHQ-9	• PHQ-9
• Sociodemographic Questionnaire.	• GRID-HAMD-17	• SCID	• SCID
• Questionnaire for Non-Participants – reasons for not taking part.	• PHQ-9	• GRID-HAMD-17	• GRID-HAMD-17
	• GAD-7	• EQ-5D-5L	• EQ-5D-5L
	• WSAS	• SF-12v2 MH Enhanced	• SF-12v2 MH Enhanced
	• CSRI (baseline and demographics)	• WSAS	• WSAS
	• EQ5D-5L	• Adapted CSRI (employment)	• Adapted CSRI (employment)
	• SF12 v2 MH Enhanced	• Questionnaire for Research Evaluation	• Questionnaire: Prior Experience with Psychological Treatment
	• Toronto Alexithymia Scale	• CSQ-8	
	• Therapy Attitude Form	• Questionnaire Therapy Feedback	
		• Questionnaires for Non-Completers	
		• Change Interview (sub-sample)	

In addition to the primary and secondary outcome measures described above, we will also collect additional data to help us to assess the feasibility of the study and describe potential moderators. The measures used to collect these additional data include: a Sociodemographic Questionnaire for participants; a Questionnaire for Non-Participants about their reasons for declining participation; Structured Clinical Interview for DSM Disorders (SCID) interview (First et al. 1997); the Client Service Receipt Inventory (CSRI) (Chisholm et al. 2000); the Toronto Alexithymia Scale (Bagby et al. 1994); a Therapy Attitude Form to assess their prior views about therapy; a Questionnaire for Research Evaluation to assess views about the research process; Client Satisfaction Questionnaire version 8 (CSQ – 8) (Larsen et al. 1979); a Questionnaire for Non-Completers of the intervention to assess their reasons for non-completion; a Questionnaire for Feedback about their Therapy; a Change Interview with a subsample; and a Questionnaire about their Prior Experience with Psychological Treatment.

### Statistical power

#### 6.1 Sample size

Fifty participants (two groups of 25) will be randomised to estimate recruitment, adherence, and retention rates, as well as the variance of outcome measures. This will provide estimates, with adequate precision to help design future studies, of the recruitment rate (e.g. if 100 people are offered participation in the study, the recruitment rate will be 50% with a 95% confidence interval (CI) of 40-60%) and the proportion followed-up at 6 months (e.g. if 40 participants are retained in the study at 6 months, the retention rate of 80% will have a 95% CI of 67-89%). The study is not designed to have power to detect differences in outcomes between groups. If 40 participants provide outcome data (20 per group) the study would only be able to detect a mean between-group difference of 0.91 standard deviation (SD) units with 80% power. Since both interventions are expected to have some benefits, a between-group difference of this magnitude is unlikely. Within groups, however, there will be 80% power to detect a mean pre-post change of 0.66 SD units in each group separately, or a mean change of 0.45 SD units in the two groups combined; note that these effect sizes relate to the SD of within-group changes, which are likely to be smaller than the SD of a single measurement. The study is likely, therefore, to detect moderate improvements in outcomes over time within groups; these estimates will help inform the design of a larger, definitive study.

### Participants and their recruitment

Fifty people with the diagnosis of persistent sub-threshold depressive symptoms and mild depression will be recruited at five general practices in Glasgow, Scotland. Eligibility to enter the trial will be confirmed at the baseline assessment by the researcher.

Identification of participants and screening for eligibility will occur in two stages:

(i). Participants will be initially screened at the GP practice by completing the self-report measure PHQ-9. Participants scoring 5–18 on the PHQ-9 (i.e. mild or moderate low mood) will be potentially eligible for the study. The GPs in the participating surgeries will be asked to screen the list of potentially eligible participants identified in their surgery to exclude anyone who meets the exclusion criteria for the study, or who they consider is unable to take part in either of the interventions.

(ii). The potentially eligible participants identified in the initial screening and not screened out by the GP, will attend the baseline assessment with a researcher. Eligibility to enter the trial will then be assessed by the researcher using a structured clinical interview (SCID).

### Inclusion and exclusion criteria

The inclusion criteria are: written informed consent obtained; aged ≥ 16; scoring 5-18 on the Patient Health Questionnaire (PHQ-9) (i.e. mild or moderate low mood) at screening; screened positive for persistent (i.e., > 6 months) sub-threshold depressive symptoms or mild depression using the SCID; and capable of taking part in research procedures. Exclusion criteria are: alcohol/substance dependence; receiving other psychological intervention; bipolar disorder; bereavement as presenting issue; Post-Traumatic Stress Disorder (PTSD); cognitive impairment; unable to understand, speak, read or write in English; Terminal illness; and unable to take part in any of the interventions.

## Ethical considerations and consent

The study received a favourable ethical opinion from Greater Glasgow and Clyde Ethics Committee REF: 12/WS/0173.

Patients’ informed consent will be obtained for each of the three stages of participation in the trial:

1. Initial screening
2. Assessment of eligibility to take part in the trial
3. Participation in the randomised part of the trial

(1) When patients arrive in the GP surgery, they will receive a pack with study information from the surgery’s receptionist or, where automated check-in systems are in place, the researcher. The pack will contain: short leaflet information, study information sheet, demographic questionnaire, PHQ-9, and initial consent form to retain this screening information (part 1 of consent form). A researcher may be present in the surgery to answer questions from patients. Patients will receive the researcher’s contact information and will be invited to contact the researcher if they have any questions about the research. Patients will have the option of taking materials away along with a reply paid envelope to return them by post or to return the completed questionnaires and consent form to a box at the practice. Care will be taken not to coerce into completing the screening questionnaires and signing the consent form for retaining this screening information. The receptionist and researcher will be instructed not to exert any pressure on the patients to return the questionnaires completed and when the researcher is present in the surgery he or she will answer questions about the study only if approached by the potential participant.

Potentially eligible participants identified by the initial screening, and not screened out by the GP, will then be contacted and invited to attend an appointment with a researcher. Those patients will receive a letter confirming the appointment which will include the full trial information leaflet for them to read. There will usually be a minimum of 48 hours between receiving the information leaflet and the appointment with the researcher. At this appointment, the researcher will take the participant through the information sheet and the patient will be given the opportunity to ask any questions they have. At this appointment:

(2) All patients will be asked to give written informed consent to be assessed for their eligibility to take part in the trial (part 2 of consent form) and

(3) If they are eligible to take part in the trial, they will be asked to give written informed consent for their participation in the trial (part 3 of consent form).

We will recruit participants in primary care; patients will therefore not be detained under the Mental Health Act and we will only recruit patients who have the capacity to give informed consent.

Participants will be made aware at the outset that participation is voluntary and that they are free to leave the study at any time without their standard care being affected.

### Randomisation

At the end of the baseline assessment, all eligible and consenting participants will be randomly allocated by the researcher to one of the two treatments through a remote telephone randomisation system, after entering the necessary baseline participant information. Randomisation will be by means of a computer-generated code. Use of an automated telephone randomisation system will ensure concealment of allocation. Participants will be randomised individually in blocks of length 4, stratified by practice to balance practice level effects. The researcher will inform (eligible and consenting) patients of the outcome of randomisation at the end of the baseline interview.

### Withdrawal of subjects

The interventions are both widely used within primary care in the NHS. Any potential risks are minimal. All participants for the duration of the trial will continue to be under the care of their GP who will be able to monitor for any adverse effects. In addition, a policy for responding to expressions of suicidal plans has been formulated. If at any time the researcher believes that there is a significant suicide risk with a patient who is participating in the study, which has not been communicated to their GP, the researcher will ask the patient for their consent to pass this information on to their GP. If the patient withholds permission for their GP to be notified, the researcher will consult the appropriate clinician (or nominated deputy) at the research site. This person will examine the patient’s data and, if it is considered necessary, will assess the patient. If it is concluded that there is a significant risk, the patient’s GP will be notified without the patient’s consent. However, the nominated clinician would contact the GP without first assessing the patient him/herself if the situation was urgent, again with or without the patient’s consent. The need to break confidentiality in situations where there was significant concern about harm to the individual (or others) will be explained in the patient information leaflet.

If a participant withdraws from the study, no further data will be collected. All data collected up to the point of withdrawal will be retained. However, the individual’s personal details would be deleted from any computerised trial records thus anonymising the retained data.

### Interventions

Patients who are eligible for the study will be randomised to receive either Person-Centred Counselling (PCC) or Low-Intensity Cognitive Behavioural Therapy (LI-CBT).

### Person-Centred Counselling (PCC)

Patients randomised to PCC will be offered eight weekly sessions of person-centred counselling delivered by qualified counsellors (minimum diploma-level training in PCC). Counselling sessions will last 50 minutes and will take place at the GP surgery or a university-based research clinic, whichever is more convenient for the participant. Prior to the trial, counsellors will receive brief refresher training in PCC, following a therapy manual designed specifically for this trial based on the Skills for Health competence framework for Humanistic Psychological Therapies (UCL 2009). All sessions will be audio-recorded with participants’ consent and a random selection will be checked for therapists’ adherence/competence by independent researchers/practitioners using the Person-Centred and Experiential Psychotherapy Scale (Freire et al. 2014); counselors who fall below the minimum adherence cut-off of 3.5 will be given feedback and additional supervision.

### Low-Intensity Cognitive Behavioural Therapy (LI-CBT)

Patients randomised to LI-CBT will receive a range of written CBT self-help resources (Williams and Chellingsworth 2010) which are supported by an optional linked online support course at http://www.llttf.com/. A DVD of the online support materials will be made available to participants without access to the Internet. Participants will receive telephone support over a series of up to eight support sessions lasting 20 – 40 minutes. Guidance will be delivered by trained support staff working with the charity, *Action on Depression Scotland*. Support sessions will follow a “Plan, Do, Review” structure which offers structured yet personalised support (Williams 2007). All sessions will be audio-recorded with participants’ consent and a random selection will be checked for support staffs’ adherence/competence by independent researchers using the Guided CBT Rating Scale^31 (pg179)^. This ensures that discussion is focused on introducing and supporting the book-based resources and that delivery is provided in an engaging and supportive way.

### Data analysis

The Robertson Centre for Biostatistics, part of the Glasgow Clinical Trials Unit, a fully registered UK CRN Clinical Trials Unit, will manage and analyse trial data. All statistical analyses will be conducted according to a pre-specified Statistical Analysis Plan, to be agreed by the Trial Management Group. Analyses will be largely descriptive, though baseline-adjusted linear regression models (or other regression models, as appropriate given the distribution of each outcome variable) will be used to estimate intervention effect differences with 95% confidence intervals, and to assess associations between potential moderator variables and outcome measures.

### Trial governance

#### Routine management of trial: Trial Management Group

The trial will be coordinated by the Trial Management Group. The Trial Management Group will comprise all co-investigators from all 3 sites. The role of the group is to monitor all aspects of the conduct and progress of the trial, ensure that the protocol is adhered to and take appropriate action to safeguard participants and the quality of the trial itself. The Trial Management Group will meet on a monthly basis.

### Trial steering committee

The Trial Steering Committee (TSC) will be established in accordance with MRC guidelines and will include a lay member (Medical Research Council 1998). The TSC will provide overall supervision of the trial and ensure that it is being conducted in accordance with the principles of Good Clinical Practice and the relevant regulations. The TSC will:

- agree the trial protocol and any protocol amendments
- provide advice to the investigators on all aspects of the trial
- have members who are independent of the investigators, in particular an independent chairperson.

Decisions about continuation or termination of the trial or substantial amendments to the protocol will be the responsibility of the TSC.

## Discussion

The NICE guidelines for depression state that “Psychological treatments are an important therapeutic option for people with sub-threshold symptoms and mild depression” (National Institute for Clinical Excellence 2009). They go on to indicate that low-intensity cognitive and behavioural interventions have the best evidence base but even for these interventions the evidence is limited and there is uncertainty about longer-term outcomes.

The evidence base is even less well established for counselling and there is also a lack of evidence about longer term outcomes for this treatment modality. Before a case can be made to provide these treatments in the National Health Service, a randomised controlled trial reporting short and medium-term outcomes (including cost-effectiveness) is required, as NICE has recommended. They have further indicated that such a trial should look at the reproducibility of the treatment model; training and supervision of those providing interventions; observer and patient-rated assessments of improvement; an assessment of the acceptability of the treatment options; clinically important effects using a non-inferiority design; and mediators and moderators of response. This feasibility/pilot study is the first step towards informing a large scale RCT that addresses these requirements.

Recruitment is a challenge in studies of depression and difficulties in recruiting patients into trials are well-documented (Woodford et al. 2011). It is, therefore, important to determine likely recruitment and retention rates for this group before embarking on a full-scale trial. This pilot study will describe the recruitment, adherence and retention rates at six months from baseline.

## Trial status

Ongoing.

## Authors’ information

EF, BSc, MSc, PhD - Lecturer in Counselling.

JM - MBChB, MSc, PhD, FRCGP – Professor of General Practice.

CW – MBChB BSc MD, FRCPsych, FBABCP – Professor of Psychosocial Psychiatry.

MC - DPhil CPsychol AcSS FBACP AFBPsS -- Professor of Counselling Psychology.

RE - PhD FAPA – Professor of Counselling, Professor Emeritus Clinical Psychology.

AM – BSc, MSc, PhD – Reader, Assistant Director of Biostatistics.

AW – BSc, MSc, PhD – Economist.

DH – BArts Sc – Research Assistant.
